# “We Need to Address the Trauma”: School Social Workers′ Views About Student and Staff Mental Health During COVID-19

**DOI:** 10.1007/s12310-022-09512-7

**Published:** 2022-02-28

**Authors:** Kate R. Watson, Gordon Capp, Ron Avi Astor, Michael S. Kelly, Rami Benbenishty

**Affiliations:** 1 Department of Social Welfare, Luskin School of Public Affairs, University of California, Los Angeles, 3357 PAB, Box 951656, Los Angeles, CA 90095-1656 USA; 2 Department of Social Work, California State University, Fullerton, Fullerton, CA 92831 USA; 3 Luskin School of Public Affairs and School of Education and Information Studies, University of California, Los Angeles, Box 951656, Los Angeles, CA 90095-1656 USA; 4 School of Social Work, Loyola University Chicago, Chicago, IL USA; 5 The Hebrew University of Jerusalem, Jerusalem, Israel; 6 Universidad Andrés Bello, Santiago, Chile

**Keywords:** COVID-19, Schools, Social work, Trauma-informed, Mental health

## Abstract

The COVID-19 pandemic and subsequent school disruptions shined a spotlight on the mental health needs of young people, and the importance of schools and school social workers (SSWs) in attending to those needs. This study sought to understand SSWs’ views about mental health and trauma in relation to the pandemic and schools reopening. Data came from written responses to open-ended questions on a national survey of SSWs during June–July 2020 (Kelly et al., 2021; Watson et al., 2022). In the national survey, 450 SSWs responded to open-ended questions, providing 115 single-spaced pages of detailed qualitative comments. A unified conceptual model for a trauma-informed school was created by integrating components suggested within the literature. This conceptual model was then used to generate a theory-based coding schema. Responses mapped well onto the conceptual model. Major themes included recognition of COVID-19 and 2020 social unrest as a unique period and potentially traumatic experience; the need for a trauma-informed school response; the challenges of addressing all facets of safety during the pandemic; and the essentiality and difficulty of maintaining school-based relationships during school closures and remote learning. Results demonstrated that SSWs used and discussed key components of a trauma-informed approach. Respondents offered several recommendations for implementing trauma-informed approaches during and after the pandemic, many of which required collaboration from other school staff. Findings provide empirical support for a unified school model that integrates components of a trauma-informed approach found in the literature. We make recommendations for interpersonal, organizational, and policy adaptations schools can take to become more trauma informed.

## Introduction

The COVID-19 pandemic and subsequent school disruptions have had a profound impact on school-age children, families, and school staff. Numerous empirical studies report increased stress and mental health difficulties among children, youth, and adults due to the fears of COVID-19 (e.g., Hatzichristou et al., 2021; Karaman et al., 2021; Murata et al., 2021). For instance, a systematic literature review conducted by Jones et al. (2021) examined 16 quantitative studies (40,076 participants) and concluded that globally, adolescents of varying backgrounds experienced higher rates of anxiety, depression, and stress and had a higher frequency of alcohol and cannabis use during the COVID-19 pandemic. These problems are compounded by the fact that children and adolescents rely to a large extent on school mental health services that were overwhelmed and interrupted during the pandemic (Duong et al., 2021; Lee, 2020; Rusch et al., 2021). Several U.S. professional associations also recently declared a national state of emergency in child and adolescent mental health (American Academy of Pediatrics [AAP], 2021). In one national survey of 1275 school social workers (SSWs; Kelly et al., 2020a, 2021), mental health services were identified as the largest unmet need of students and their families during the early phase of the pandemic. More than 75% of respondents indicated that the majority of families served by their schools had unmet needs for mental health services. Further, 65% of SSWs thought that poverty and 60.5% believed mental health problems were compounding the pandemic experience for students and families. SSWs reported that families with students attending low-resourced schools had higher needs for basic supports (e.g., for food, health care, and housing) and other services (e.g., mental health, disability, or tutoring support; Watson et al., 2022). Findings highlighted some of the many challenges that children and families have faced during the pandemic as a result of increased family stress due to school closures, loss of jobs or housing, social isolation, and the possibility of traumatic loss or grief. There are also widespread concerns that intense family stress contributed to higher rates of domestic violence and child maltreatment during the pandemic, including unreported events (Evans et al., 2020; Swedo et al., 2020; Usher et al., 2020). All of the above were unfolding amidst civil unrest and a nationwide racial reckoning (Drew, 2020).

As schools reopened for in-person or hybrid instruction, they were tasked with responding to the heightened mental health needs of students, families, and staff resulting from potentially traumatic experiences during the pandemic. Given recent calls to address students’ pandemic-related traumatic experiences coupled with newly available funding for such purposes (Jones, 2020, 2021), it is essential that we understand what frontline providers of school mental health like school social workers (Kelly et al., 2015; National Association of Social Workers [NASW], 2010) think about the trauma and related needs of students and staff, and their recommendations for appropriate school trauma responses.

In our initial inductive analysis of qualitative responses to the aforementioned study (Kelly et al., 2020a; Capp et al., 2021), we noticed that a sizable proportion of school social workers specifically utilized the word “trauma” or mentioned concerns about meeting students’ mental health and trauma-related needs in their written responses to open-ended survey questions. The study reported in this article is guided by earlier findings (see Kelly et al., 2020a; Capp et al., 2021) and seeks to better understand how SSWs were thinking about trauma and their role in providing care to students and staff during COVID-19, through their own detailed written responses. This study also proposes a new overarching conceptual model that integrates disparate components of a trauma-informed school approach in the research literature and then assesses if the themes suggested by SSWs map onto the conceptual framework during the pandemic. The research questions guiding this study are: what are school social workers’ perspectives about trauma in relation to the COVID-19 pandemic? What do SSWs highlight as the trauma-related needs of school constituents during and after the pandemic? How do school social workers’ views relate to existing models for trauma-informed schools? This article considers how proposed components of a trauma-informed school (e.g., recognizing and responding to trauma, ensuring safety, and promoting healthy relationships) relate to the activities of SSWs during the pandemic. The study goal is to empirically test a conceptually unified set of components for trauma-informed schools and to inform current educational policy and social work practice.

### Trauma-Informed Schools (TIS): A Synthesis of Components Within the Literature

In the last two decades, there has been interest from mental health practitioners, intervention developers, and policymakers in adapting organizational policies and practices to better support individuals with trauma histories (Harris & Fallot, 2001; Maynard et al., 2019). Because a consensus-based model for a whole-school, trauma-informed approach does not exist, the first author reviewed available models within the trauma, education, and other literature to identify different components that have been proposed by multiple models. A synthesis of these models suggested seven possible components for a whole-school, trauma-informed approach. Table 1 outlines the components: (1) understanding the prevalence of trauma; (2) recognizing trauma symptoms; (3) responding to trauma through trauma-informed practice and policy adaptations; (4) ensuring safety; (5) developing and sustaining positive, school-based relationships; (6) adopting strategies for culturally responsive care; and (7) seeking to avoid retraumatization (see Cole et al., 2013; Harris & Fallot, 2001; Hopper et al., 2010; Milwaukee Public Schools [MPS], 2020; National Child Traumatic Stress Network [NCTSN], 2017; Substance Abuse and Mental Health Services Administration [SAMHSA], 2014; University of Maryland [UMD], n.d.; Wolpow et al., 2009).

**Table 1:** Components of a trauma-informed approach for schools identified in the literature

Component	Models including component
Understanding the prevalence of trauma in society	Cole et al. (2013), Harris and Fallot (2001), Hopper et al. (2010), MPS (2020), NCTSN (2017), SAMHSA (2014)
Recognizing trauma reactions in all members of the school, including oneself	Harris and Fallot (2001), Hopper et al. (2010), MPS (2020), NCTSN (2017), SAMHSA (2014), UMD n.d., Wolpow et al. (2009)
Responding to trauma through policy and practice adaptations	Cole et al. (2013), Harris and Fallot (2001), Hopper et al. (2010), MPS (2020), NCTSN (2017), SAMHSA (2014), UMD n.d.
Ensuring physical, emotional, and psychological safety	Cole et al. (2013), Harris and Fallot (2001), Hopper et al. (2010), NCTSN (2017), SAMHSA (2014), Wolpow et al. (2009)
*Relationships that are…*	
Trust-based	Cole et al. (2013), Harris and Fallot (2001), Hopper et al. (2010), MPS (2020), SAMHSA (2014), UMD n.d., Wolpow et al. (2009)
Collaborative	Cole et al. (2013), Harris and Fallot (2001), MPS (2020), NCTSN (2017), SAMHSA (2014), UMD n.d., Wolpow et al. (2009)
Empowering and giving voice to all constituents	Harris and Fallot (2001), Hopper et al. (2010), MPS (2020), SAMHSA (2014), UMD n.d., Wolpow et al. (2009)
Cultural responsiveness, including recognizing constituents in their broader ecology and the historical trauma of marginalized groups	Hopper et al. (2010), MPS (2020), NCTSN (2017), SAMHSA (2014), UMD n.d., Wolpow et al. (2009)
Avoiding retraumatization	MPS (2020), NCTSN (2017), SAMHSA (2014)

*Understanding the prevalence of trauma and recognizing trauma symptoms*, the first two components of a trauma-informed approach identified in the literature, underscore the importance of school staff being aware of the impact of childhood trauma on academic achievement and learning and having access to knowledge and resources to identify trauma symptoms in students, other staff, and themselves (Cole et al., 2013; Harris & Fallot, 2001; Hopper et al., 2010; NCTSN, 2017; SAMHSA, 2014). Universal trauma training is typically recommended to meet these first two goals (Cole et al., 2013; MPS, 2020; NCTSN, 2017). Some models for a trauma-informed organizational approach also recommend universal screenings to detect individuals with trauma histories or symptomatology (Harris & Fallot, 2001; Hopper et al., 2010; NCTSN, 2017). School-based trauma screenings are controversial, however. Two school-focused models specifically reject universal screenings as privacy violations that may stigmatize students (Cole et al., 2005, 2013; Wolpow et al., 2009).

Component three, *responding to trauma through practice and policy adaptations*, reflects a need for school procedures and policies to support trauma survivors rather than require survivors to adapt to a typical school environment (Cole et al., 2013; Harris & Fallot, 2001; Hopper et al., 2010; MPS, 2020; SAMHSA, 2014; UMD, n.d.). Cole et al. (2013) caution that solely adopting a trauma-related treatment does not make a school trauma informed, and that all policy or procedural adaptations must allow flexibility for educators and administrators to match a school’s needs to identified solutions. Frequently recommended policy changes include the elimination of zero-tolerance discipline, whereby a child may be expelled after one school offense, and other exclusionary practices such as office discipline referrals or suspensions (Cole et al., 2005; NCTSN, 2017). With regard to instructional practices, teachers can establish routines to enhance predictability (Cole et al., 2005), implement systems to notify children in advance of changes (e.g., a bell that chimes before class transitions), and establish a quiet space to which students can retreat when overstimulated (NCTSN, 2017).

Component four, *creating a sense of physical, psychological, and emotional safety*, is included in several conceptualizations for a trauma-informed approach (e.g., Harris & Fallot, 2001; Hopper et al., 2010). Addressing a school’s physical safety refers to the creation of a warm, welcoming environment that allows adequate personal space; has appropriate lighting, security, and accessibility; and is free from physical hazards (Elliott et al., 2005; Levenson, 2017). Psychological and emotional safety result when an environment is free from potentially triggering materials and curricula, and interpersonal interactions are respectful, consistent, and adhere to privacy and confidentiality policies (Elliott et al., 2005; Harris & Fallot, 2001; Hopper et al., 2010; Levenson, 2017). A trauma-informed school should strive to ensure that all members of the school community feel safe and welcomed.

*Positive relationships*, component five of the trauma-informed school model, are highlighted as important for a variety of reasons across models for a trauma-informed approach (e.g., Cole et al., 2013; Harris & Fallot, 2001; Hopper et al., 2010; MPS, 2020; NCTSN, 2017; SAMHSA, 2014; UMD, n.d.; Wolpow et al., 2009). One reason for the focus on relationships is that trauma often occurs in relationships, and future consistent, caring relational experiences can help heal past trauma (Dods, 2013). Positive relationships are facilitated by open, transparent decision making; collaborating and minimizing power differentials; and permitting staff and students to make decisions about their priorities (Cole et al., 2013; Harris & Fallot, 2001; Hopper et al., 2010; MPS, 2020; SAMHSA, 2014; UMD, n.d.; Wolpow et al., 2009).

Component six, *cultural awareness and responsiveness*, includes recognizing individuals in the context of their broader ecology, e.g., family situation, neighborhood, and membership in religious, ethnic, or racial groups, as well as acknowledging the collective trauma of historically marginalized or oppressed populations (Hopper et al., 2010; MPS, 2020; NCTSN, 2017; SAMHSA, 2014; UMD, n.d.; Wolpow et al., 2009). Cultural responsiveness does not require in-depth knowledge of all racial, ethnic, and religious backgrounds, but instead a willingness to ask questions and seek to understand a person’s cultural context (Elliott et al., 2005). Teachers can demonstrate cultural responsiveness by asking students about their backgrounds and culture, by incorporating diverse perspectives in classroom materials and assignments, and by encouraging the school to recognize a variety of holidays. It is also important for staff to educate themselves about race and racism, and the ways that their own implicit biases may affect their perceptions of and interactions with students (Astor et al., 2021).

Component seven, based on the final phrase in SAMHSA’s (2014) definition of a trauma-informed organization, is *avoiding retraumatization*. Although additional steps are not required to avoid retraumatization, the goal needs to be included as part of a school’s rationale for implementing the prior six components (e.g., creating a sense of physical and interpersonal safety; educating staff about trauma and its effects; adapting practices to avoid potentially traumatizing situations; and developing and sustaining positive school-based relationships; NCTSN, 2017; MPS, 2020; SAMHSA, 2014).

The integrated conceptual model for a trauma-informed school presented above served as a framework for our exploration of school social workers’ understanding of and responses to the trauma-related needs of schools, students, and staff during COVID-19 school disruptions. As frontline providers of mental health services and the professionals most likely to be tasked with implementation of trauma-informed approaches in schools, SSWs’ views of the trauma-related needs of students and staff are critical to any discussion of how schools should operate safely using a trauma-informed response during the pandemic era.

## Methods

Data for this study came from a national survey of school social workers, social work supervisors, and district heads of social work services during the months of June–July 2020. Survey instruments sought to understand, from the perspective of school social workers, the needs of PreK-12 schools, staff, and students during the COVID-19 pandemic, including what support and resources were suggested as schools reopened. Participants were recruited with requests to complete the survey through national, state, and local social work organizations, including the School Social Work Network, National Association of Social Workers, American Council for School Social Work, School Social Work Association of America, and Society for Social Work and Research. The survey link was distributed through multiple social media outlets, including Twitter and Facebook. Researchers received institutional review board approval from their respective universities, and partner organizations went through independent ethics review processes. This study focuses exclusively on the open-ended qualitative responses to the survey. Quantitative results have been published (see Kelly et al., 2021; Watson et al., 2022).

### Participant and School Characteristics

Respondents (*N *= 450) primarily identified as female (91.6%). Males accounted for 7.3% of respondents, gender non-conforming individuals for 0.7%, and two (0.4%) chose not to disclose. Participants self-identifying as White accounted for 73.8% of responses; as Black, 12.2%; Hispanic or Latinx, 10.4%; and as other races or ethnicities, 3.6%. Respondents hailed from 43 U.S. states, with the majority based in Illinois (*n* = 119), Connecticut (*n* = 81), Michigan (*n* = 77), and California (*n* = 40). Most respondents (90.9%) reported serving as school social workers, and remaining respondents were either social work supervisors (3.6%) or district heads of social work services (5.3%). Participants were very experienced; more than 30% had 20 or more years of service and an additional 31.8% had 11–20 years of service. Only 1.8% reported less than a year of service, 8.0% reported 1–2 years, 12.4% 3–5 years, and 14.9% 6–10 years. Participants were asked to report on the primary school with which they worked. Many reported working across multiple schools: 73.1% reported working in middle or junior high schools, 69.8% in elementary schools, 50.0% in high schools, 38.4% in preschools or child-care settings, and 26.9% in alternative schools. In the schools they served, respondents reported that on average, 60.4% of students qualified for free/reduced lunch, 50.2% were from historically marginalized ethnic and racial groups, 14.2% dropped out, and 56.0% entered college. A table of participant/school characteristics has been published (see Capp et al., 2021).

### Qualitative Questions Asked

In the national study (see Capp et al., 2021; Watson et al., 2022), approximately 35% of participants responded to the open-ended questions (*N* = 450). A follow-up with respondents provided a total of 115 single-spaced pages of rich and detailed qualitative comments. This study focuses exclusively on the 450 participants that answered open-ended questions. Open-ended questions asked participants to share:

1. Any comments, suggestions, or thoughts that could help us better understand your current experiences as a school social worker or supervisor, and any practice suggestions that could benefit other social workers, professional leaders, or relevant policymakers.
2. What recommendations for reopening the education system do you have for your district (e.g., policies, procedures)?
3. What are the biggest needs and challenges you have identified among students and their families during the pandemic, and what support/resources do you need to address them for the new school year?
4. What are the biggest challenges and obstacles you are facing personally and professionally? What policies (school, district, or state-level) are needed to support your ongoing work?
5. What supports did you receive from others (e.g., school and district staff and leadership, community, etc.) that were helpful for your work?
6. What support/resources did you provide for school staff members and leadership?
7. What changes and innovations in your practice have you made that might help other social work professionals? For instance, were you able to increase student engagement while schools were closed? Were there other innovations that were helpful?

Data analysis involved both deductive and inductive elements, which may be referred to as a blended approach (Linneberg & Korsgaard, 2019). Deductive elements (Miles et al., 2018) provided organizational structure to the coding process and helped ensure thematic relevance to a conceptual framework for a trauma-informed school. During analysis, we also allowed additional themes to emerge from the data based on participant responses. The blended approach (Linneberg & Korsgaard, 2019) allowed for an in-depth analysis of the extent to which SSWs’ responses and actions during the pandemic connected to a conceptual framework for a trauma-informed school and enabled identification of potential gaps and implications for the future development of such approaches.

Analyses were partially based on the conceptual framework for a trauma-informed school presented in the introduction of this article. The conceptual framework included a review of available models in the literature (see Tables 1 and 2). The seven identified components of a trauma-informed school served as the overarching umbrella for our coding schema (Miles et al., 2018). During coding, we allowed for new themes to emerge and for additional themes that elaborated on the meaning of these concepts for our participants. Within the extant literature on trauma-informed schools and organizations, the first two conceptual components, understanding the prevalence of trauma and recognizing trauma symptoms, are often discussed as one component. Thus, based on the literature, we combined these two components into a single code. Similarly, component three, responding to trauma, is often discussed in two ways. One way it is discussed is as an organizational factor and the other way is as a direct-practice component. Thus, based on this divide in the literature, we subdivided the third conceptual component into organizational-level responses and direct service responses. Codes for avoiding retraumatization; safety; relationships; and cultural responsiveness were translated directly from the literature. These seven codes served as the basis for our coding frame.

**Table 2:** Comparison of trauma-informed components to coding schema and to emergent themes

Component	Coding schema	Emergent themes
1. Understanding the prevalence of trauma	Realizing and recognizing trauma	(1) Recognition of COVID-19 and 2020 social inequity as a potentially traumatic experience requiring an appropriate response
2. Recognizing trauma reactions		
3. Responding to trauma through policy and practice adaptations	• Responding to trauma: direct practice • Responding to trauma: organizational	(2) The need for a trauma-informed response, including: a) Universal trauma training for staff b) Increased organizational capacity and resources to address mental health c) Prioritizing social emotional needs above academics upon return to school d) Supporting staff with self-care and STS resources e) Integration of SSW voices in administrative responses f) Recognition of SSWs’ socioecological perspective
4. Ensuring physical, emotional, and psychological safety	Safety	(3) Challenges of addressing all facets of safety
5. Relationships that are trust-based, collaborative, empowering	Relationships	(4) Essentiality and difficulty of maintaining school-based relationships
6. Cultural responsiveness	Cultural responsiveness	(1) Recognition of COVID-19 and 2020 social inequity as a potentially traumatic experience requiring an appropriate response
7. Avoiding retraumatization	Avoiding retraumatization	

Participant responses were imported into NVivo (2020) for analysis. All responses were independently coded by two members of the research team who met regularly to ensure consistency in application of the conceptual framework and coding schema and congruence among emerging themes. An initial set of themes indicating how SSWs viewed trauma and trauma-informed school approaches was developed and discussed with all members of the research team. To enhance rigor, team members took part in regular debriefing, including discussions of positionality and subjectivity in the research process (Padgett, 2011). All five members of the research team are professors or doctoral students in social work departments, and four have direct-practice experience in schools.

## Findings

Four primary themes related to the conceptual framework for a trauma-informed school emerged from SSWs’ written responses. Themes included: (1) recognition of COVID-19 and 2020 social inequity, racism, and political strife as a potentially traumatic experience; (2) the need for a trauma-informed response from schools; (3) the challenges of simultaneously addressing all facets of safety during a pandemic; and (4) the essentiality and difficulty of maintaining school-based relationships during school closures and remote learning. Table 2 maps components of a trauma-informed approach identified in the literature to our coding schema and emergent themes.

### Theme 1: COVID-19, Racism, and Political Strife as a Potentially Traumatic Experience

SSWs recognized the COVID-19 pandemic and social unrest of summer 2020 as historic moments and potentially traumatic experiences that could have significant long-term impact on students and families. For example, one SSW said, “COVID-19, and the recent events shining light on racism, is impacting our entire city.” Another explained the imperative to attend to social inequities that impact high-need groups:

The students least likely to have access [to remote instruction] come from areas of greater poverty, are more likely to be minority students, and many are recent immigrants or undocumented. Many of these families and students already face many barriers and have been affected by job loss and financial strain as well as being more likely to have family members sick with or dying from Covid-19. And now they are even further behind their peers in school. This is why people are protesting in the streets, because our systems are stacked against our students and families. It needs to change!

Looking toward a return to in-person instruction, one SSW predicted, “The biggest challenge will be addressing trauma reactions (aggressive behavior, sleep disruptions, increased anxiety, etc.), and helping students to re-adjust to the structure of school.”

SSWs also made suggestions for school response to the racial reckoning. One shared:

I think we need to strengthen our staff’s capacities in diversity, equity, and inclusion. We are not only facing a health pandemic, but we are also seeing a huge racial movement right now and I think we need to be very mindful of how we are talking about this with students when we return and ensure that we ARE talking about it with our students.

Solutions offered by SSWs included “incorporation of intentional, measurable actions of anti-racism,” “cultural and racial sensitivity training to combat ignorance and injustice,” and “more educators with a variety of cultural and [ethnic] backgrounds who love kids.” One SSW suggested a need for “everyone to understand and be more sensitive to what daily traumas and life conditions many of the students in the urban school environment experience, and the historical implications that have defined these experiences.”

SSWs’ responses illustrated that they viewed the early phase of the pandemic and 2020 civil rights movement as a potentially traumatizing experience for students and families. SSWs also indicated that they believed schools needed to address the trauma students faced during this period, and that their responses should be trauma informed. SSWs’ responses within this theme illustrate two components of the trauma-informed conceptual framework synthesized from the literature: component one, *understanding* the prevalence of trauma, and component six, *providing* culturally responsive care (see Table 2).

### Theme 2: The Need for a Trauma-Informed Response to the Historic Moment

SSWs’ responses focused on the need for schools to both recognize the potential trauma resulting from the present moment and respond in a trauma-informed manner. SSWs made numerous recommendations for school-based responses to trauma, including: (1) universal trauma training for staff, (2) increased organizational capacity and resources to address student and staff mental health, (3) prioritizing social-emotional needs above academics upon return to school, (4) supporting staff with self-care and secondary traumatic stress (STS) resources, (5) integration of SSW voices in administrative responses, and (6) recognition of the unique socioecological perspective SSWs offer schools. These recommendations relate to components two and three of a trauma-informed school approach, *recognizing* trauma signs and symptoms and *responding* to trauma by adapting school practices and policies (see Table 2).

#### A Call for Universal Trauma Training to Prepare Staff to Respond to Heightened Needs of Students and Staff

To help school staff recognize and respond to traumatic experiences and trauma reactions, SSWs pointed to a need for universal trauma training. Often this recommendation was made in conjunction with the recognition that teachers and administrators had been surprised by or unprepared for the number of students who did not access remote instruction during school closures. One respondent described, “We spent a great deal of time explaining to admin and staff what a pandemic/community trauma does to our brain and how we should not expect school and academics to be business as usual.” In promoting a need for training, another SSW said, “Teachers and other education professionals need to know how trauma impacts learning and understand that the pandemic’s impact on [students’] lives and that of their families is most likely traumatic.” A third respondent noted, “I have recommended to my own school network that we train all of our staff in mental health first aid so that all adults in the building can recognize the signs of trauma and distress in our students.” Some SSWs were already engaged in helping their school implement trauma training. For example, one wrote, “Our district is prioritizing SEL/trauma training…upon return to school. Staff will be trained and they will facilitate classroom lessons that focus on addressing trauma issues that may have occurred during the shutdown.” SSWs’ calls for trauma training relate both to the second and third components of a trauma-informed approach, *recognizing* trauma symptoms and *responding* through practice and policy adaptations. This subtheme also highlights that SSWs saw summer 2020 as a potentially traumatic time requiring an appropriate school response.

#### Increased Organizational Capacity and Mental Health Resources to Address Student and Staff Needs Related to COVID-19 and Issues of Social Injustice

SSWs’ responses emphasized a need for additional mental health resources during and after the pandemic. One explained, “I think we have not fully considered the impact of potential trauma for staff or the students, nor have we provided staff with resources, policies, supports, and training for their own mental health.” A second stated, “We need to address the trauma and provide all the tools everyone, and I mean everyone, who works in a school setting [needs]. This a new normal we need to create.” In keeping with SSWs recognition of the increased need for mental health and trauma-related support as a result of the pandemic, SSWs highlighted capacity issues. One SSW said, “More social workers [are needed] to support the ever-growing need of mental health and the trauma that this [pandemic] has created. Not only will we be dealing with the students and families, but the staff as well.” A social work supervisor added, “Caseloads have to be reasonable for social workers to meet the changing needs of their students and families as well as assisting with the mental health needs of school staff.” Overall, SSWs’ responses indicated concern about their ability to meet the heightened mental health and trauma-related needs of staff and students upon returning to school without an increase in organizational capacity. Their responses illustrate component three of a trauma-informed approach in schools, *responding* to trauma through policy and procedure adaptations. SSWs’ concerns went beyond what they could manage individually and highlighted a need for an organized, schoolwide response.

#### Prioritizing Social Emotional (SEL) Needs Above Academics upon Return to School

Another element of trauma responsiveness SSWs highlighted was a need to address social and emotional well-being upon return to school. One said, “Our team feels strongly that academics cannot be the first item of focus. Routine, structure, relationships, trust, [and] alleviating fears will be the first things that need addressing.” Another suggested that SSWs should advocate for socioemotional well-being: “It is important to continue to advocate for mental health and social emotional needs. It is ‘easy’ to focus on the academics, without thinking about the mental health and social emotional needs…these are educators, not social workers!” SSWs reported struggling to convince school leadership of the importance of meeting students’ socioemotional needs during the pandemic. For example, one said, “I'm trying to get the Administrative team and the District to recognize that just coming back to school and starting as we have every other year is not appropriate. We need to deal with the students’ [social emotional] needs first so that they can learn.” Still, other respondents expressed frustration that staff had not yet accepted the need for schoolwide adaptations in response to trauma:

Even after 20 years of educating school staff to the effects of trauma, the challenges of mental health issues, and the needs of students living in poverty, during this crisis many staff continue to not understand and refuse to make accommodations to provide additional resources and support for these students. This has been my biggest frustration and challenge during this time.

Overall, SSW responses indicated that a return to business as usual would not suffice to meet the elevated needs of students following the pandemic and 2020 racial reckoning. Responses suggest that schools must go beyond providing training about trauma to also adjust organizational expectations and policies to meet the needs of students and staff following the pandemic. Such responses illustrate component three of a trauma-informed approach in schools, *responding* to the needs of trauma survivors, and doing so from an organizational perspective.

#### Supporting Staff with Self-Care and Secondary Traumatic Stress (STS) Resources During and After the Pandemic

SSWs shared their efforts to support staff with self-care and STS resources, often with mixed results. One explained, “I offered self-care to teachers which was poorly received and I was spoken to by my administrators that teachers complained it was not helpful and not student focused.” Another reported difficulty balancing support for others with their own self-care: “I attempted to provide emotional support for colleagues and administrators and helped to problem solve with them… However, it was difficult as I was also going through the same stressors and unknowns that my colleagues were.” A third reported, “There has been no dialogue about secondary trauma or caregiver fatigue among those of us in the district who work in mental health. We are ALL dealing with COVID.” “To avoid losing staff,” one SSW suggested, “districts will need to consider what they can do to help teachers emotionally as they transition back into the buildings.” As can be seen in the above responses, SSWs believed that when educators and administrators addressed trauma reactions, their focus tended to be on students rather than staff. Such responses illustrate the imperative of shifting to a whole-school response to trauma instead of relegating the responsibility to one or two mental health professionals. How SSWs viewed the role of schools in *responding* to trauma illustrates component three of the blended conceptual framework for a trauma-informed school.

#### A Call for Better Integration of SSW Voices in Administrative Responses to the Pandemic, Including in Policy Development and Implementation

SSWs reported a lack of connection with administrators or district leadership during the pandemic. One said, “As always there is a disconnect between the social work providers (direct service) and the administration.” Some of this disconnect may be attributed to insufficient communication: “My total guidance [during the pandemic] was ‘do the best you can’ and then, nearly all efforts were criticized because students weren't engaging.” SSWs suggested a need for greater involvement in their schools’ and district’s pandemic response. One shared, “I think administration should work WITH social workers to develop a plan for what remote services should look like. It should be uniform, at the very least, across a district.” Another recommended, “At an administrative level, (we need to have) social work input ‘at the table’ as decisions are being made for students, staff and families.” SSWs indicated a need for a variety of policies and practice adaptations related to COVID, including “guidance from national organizations on how and when to provide social work services to families/students during each of the many phases of education (hybrid, in-class, remote),” “collaboration from social service and non-profit agencies to meet the economic needs of families,” and “clear guidelines (about COVID) for families that they can understand.”

SSWs’ requests for greater involvement in schoolwide pandemic responses and for specific policies indicate why school social workers and other mental health professionals must be engaged in decisions regarding the pandemic and reopening schools. This subtheme again illustrates component three of the conceptual framework for a trauma-informed school, *responding* to trauma through procedure and policy changes.

#### The Unique Socioecological and Organizational Perspective that Social Workers Bring to Schools

As shown above, respondents illustrated a person-in-environment lens, emphasizing the societal and familial factors impacting student and staff COVID-19 experiences. One shared:

I work in an environment where many students are removed from their community settings due to abuse/neglect, mental health challenges... seemingly intractable issues...compounded by their levels of historical poverty, lack of access to resources, and the intergenerational factors and social structures that continue to limit their progress in breaking those cycles of deficits. School social workers tend to be [among the] very few, if not the only, professionals working within the school environment that are sensitive towards and able to… highlight these types of psychosocial factors.

SSWs also indicated a need for basic resources, including food and technology to engage in remote learning, during the pandemic. One explained:

Our skills allow us to know how to function during a crisis. My role may have shifted from direct care to more basic needs outreach (food insecurity, device distribution, housing support, home visits) but there [are] still enough mental health/crisis concerns to allow for continued direct care.

Social workers’ focus on meeting the basic needs of students and their families, and their socioecological lens were supported by our quantitative findings (Kelly et al., 2021; Watson et al., 2022). This subtheme highlights the importance of involving social workers in pandemic *response* (component three) due to their focus on broader issues impacting students, families, and staff.

### Theme 3: Challenges of Simultaneously Addressing all Facets of Safety During a Pandemic

The third theme that emerged from SSW responses was a concern for how to respond to competing aspects of safety during the pandemic (see Table 2). Respondents conceptualized safety in a variety of ways: physical, psychological, and emotional. These varied definitions of safety resulted in conflicting suggestions about how best to reopen schools and support staff and students during the pandemic. Almost universally, SSWs emphasized a need for emotional safety after the pandemic. For example, one said:

Our primary focus upon return should be safety. A huge part of that is emotional—we must give students and staff time to process all that has occurred, share their concerns and fears about being back, and provide strategies for coping, reporting difficulties, etc. …If individuals are not feeling safe and heard, no learning will occur.

Social workers like this one favored returning to in-person classes quickly to ensure kids benefited from a daytime environment free from family stressors and coupled with peer and staff support:

I ache for the children who don’t have school as their safe place. Not seeing the children is hard for me. If at all possible, I’d love to see school districts brainstorm a way to safely have children in school at least half a day for half a week.

Other SSWs were concerned about the risk of infection for themselves and staff, should schools reopen in person quickly, and expressed worry that enhanced on-site safety features could scare young children or create an unwelcoming school environment:

My school social work colleagues and I are very concerned about the effects of fear and uncertainty on the youngest children we serve, in particular. While necessary for health and safety, constant use of masks, temp checks, policing around keeping distance, etc., could make school feel like an unsafe, unsupportive place, could certainly be not trauma-informed, and could cause more emotional damage to our youngest children. This should be considered in creating reopening procedures.

Still other SSWs specifically referenced the challenge of simultaneously meeting multiple definitions of safety. One said, “This is so complicated on so many different levels. Kids are ‘screaming’ to come back for social emotional support. This needs to be weighed against the physical [safety] concerns.” SSWs’ responses here illustrated the importance of *safety for all school constituents*, the fourth component of a trauma-informed approach synthesized from the literature. One respondent tied this theme directly to a trauma-informed approach: “Being a trauma-informed and educated practitioner, I believe that every effort needs to be made to keep people healthy, alive, and safe.”

### Theme 4: Essentiality and Difficulty of Maintaining School-Based Relationships During COVID-19

Throughout their responses, SSWs highlighted the importance of positive relationships among members of the school community. In keeping with our synthesis model for a trauma-informed school, we also evaluated the extent to which SSWs specifically reflected upon various elements of positive relationships found in the literature, including trust, collaboration, and empowerment (Harris & Fallot, 2001; Hopper et al., 2010; MPS, 2020; SAMHSA, 2014). As an example of how SSWs thought about school-based relationships, one shared:

The relationships are paramount, with students, families and staff. As a collaborative school that works with various populations and disabilities, we pride ourselves on the relationships we build with students and families. As we went virtual, these relationships allowed us to, in many cases, quickly engage with students and families.

Another explained, “Student healing can happen through relationships we have with our students. [In] trauma-informed care, healing happens through the relationship.” A third added, “I think this [situation] has underscored the importance of building relationships with parents. As an elementary school worker, I have had to rely heavily on the parents in order to connect with their students.” At the same time that SSWs shared the importance of staff and students connecting with peers and others in school, they noted they were struggling due to physical distancing requirements. One SSW said that both students and teachers were having difficulty “navigating the social distancing rules and wanting to spend time with their friends and interact socially, but being restricted for safety reasons.” SSWs also reported a personal sense of disconnection from school, students, and coworkers: “About one month into E-Learning, I found myself feeling anxious and depressed like I would do this forever! This was frustrating for me as I love the interaction with my students and staff, but not via a laptop.” Others shared actions they took to ameliorate the situation:

Typically, my colleagues and I support each other with [difficult] cases and this has been harder to [do] during the closure. We have all realized we are missing this collegial support/supervision to process cases and are now reaching out and talking by phone, text, or setting up a google meet with each other to get the support we need, or to brainstorm how to support a child or family. That has been very helpful!

Overall SSWs’ responses indicated a strong understanding of component five of a trauma-informed school approach: the importance of developing and sustaining positive, school-based *relationships*. The following subthemes delve into specific aspects of positive relationships typically associated with a trauma-informed approach: trust, collaboration, and empowerment.

#### The Importance of Trust when Working with Families Before and During the Pandemic

SSWs’ responses highlighted the importance of developing trusting relationships with families both prior to and during the pandemic. One claimed, “It is extremely important to create and maintain connections with students and families so in time of crisis the family trusts and reaches out to you.”

#### The Importance of Collaboration Among School Professionals During the Pandemic

SSWs also highlighted the importance and benefits of working collaboratively with others in their department or school environment (e.g., counselors, teachers, and administrators) during the pandemic. One said, “More now than ever, teamwork is essential. A little support can go a long way for staff and the families we serve.” Another praised their district’s collaboration and communication, “My school district was organized, supportive and openly communicated with all students, staff and parents. I felt appreciated by my supervisors and colleagues and we worked as a cohesive team to make what we could work.”

#### Empowerment and Giving Voice as a Characteristic of Positive School-Based Relationships During a Pandemic

SSWs mentioned empowerment or giving voice to families as part of their pandemic response. One shared, “Empowering parents/caregivers to more effectively support their children's education…could help, but the parents who most need whatever we offer are always the parents who have the most roadblocks keeping them from receiving what we have to offer.” At the same time, SSWs reported a lack of voice in their own roles. One simply stated, “There is no need to make recommendations [related to reopening] because I don’t have a voice.” Another advocated for what they wanted: “[I need] to be part of the team—I feel like my input is not wanted throughout this.”

SSWs’ responses across this theme point to the primacy of *relationships* in promoting healing and post-traumatic recovery, component five of a trauma-informed approach in schools. However, they did not address all characteristics of positive relationships across all constituents (students, staff, and families).

## Discussion

This study explored how school social workers viewed trauma and mental health in relation to the COVID-19 pandemic, and how their responses related to existing conceptual models for a trauma-informed approach in schools. SSWs’ suggestions largely supported the conceptual framework for a trauma-informed school synthesized from the literature but were specific to their role and socioecological perspective. Key themes included recognition of COVID-19 and the 2020 racial reckoning as a potentially traumatic experience, the need for a trauma-informed school response, the challenges of simultaneously addressing multiple facets of safety during the pandemic, and the essentiality and difficulty of maintaining school-based relationships during school disruptions. Recommendations deemed essential to a trauma-informed school response included universal trauma training, additional mental health resources and expanded organizational capacity, and better integration of SSW voices in administrative decisions related to pandemic response.

### Connection Between Findings and the Trauma-Informed Schools Conceptual Framework

#### All but One Component of the Conceptual Framework Reflected in Findings

As can be seen in Table 2, our findings demonstrate that SSWs were aware of the possibility of trauma associated with pandemic-related school disruptions and they recommended numerous practice and policy adaptations to alleviate such trauma. Overall, SSWs’ responses encompassed six of the seven literature-informed components of a trauma-informed school: understanding the prevalence of trauma in society; recognizing trauma reactions; responding to trauma through policy and practice adaptations; ensuring physical, psychological, and emotional safety; promoting and sustaining healthy relationships; and acting in a culturally responsive manner. The exception was component seven, avoiding retraumatization. It is clear from the analysis that SSWs sought to ensure student and staff safety and to make appropriate adaptations to their individual practices and school policies, which could contribute to avoiding retraumatization, but no respondent claimed a goal of avoiding retraumatization as part of these efforts. Their focus was instead on adapting to the trauma presumed already present or responding to the heightened needs of students and staff resulting from the pandemic. Given that the ideal of avoiding retraumatization can be served by incorporating many other components of a trauma-informed approach (e.g., ensuring safety, educating staff about trauma, adapting services commensurately, and promoting healthy relationships; Harris & Fallot, 2001; Keeshin & Strawn, 2014), it is possible that the goal is being met without SSWs being conscious of it or focusing on it specifically. At the same time, it is possible that the lack of specific reference to this goal is an oversight by SSWs. Future research is suggested to determine how SSWs view their role in relation to the avoidance of retraumatization.

#### The Current Historical Moment as a Potentially Traumatic Experience, and the Need for a Trauma-Informed Response

Findings illustrate that SSWs viewed summer 2020, the early phase of the COVID-19 pandemic and a period marked by renewed calls for civil rights and social justice for Black Americans, as a potentially traumatic time for students, staff, and families. We know certain historical periods are salient to community trauma (e.g., 9/11 and wartime; Micale & Lerner, 2001; Van der Kolk, 2015). Summer 2020 seems to be another such time. Although SAMHSA (2014) highlights that responses to trauma must be understood and addressed in context (specifically in regard to community factors), the context of time and the social norms of the period have been left out of existing models for trauma-informed approaches. We recommend that future conceptual models for trauma-informed approaches in schools integrate history and time as two of the contexts considered. Such integration can be guided by existing models for school safety and climate (e.g., Astor & Benbenishty, 2019). With schools reopening for in-person instruction, school staff and administrators will need to consider the potentially traumatic experiences students and staff may have experienced individually or collectively during the pandemic and respond appropriately to support future learning.

#### A Need for Expanded Focus Related to Cultural Responsiveness

Current conceptual models for trauma-informed schools include a value of cultural responsiveness. SAMHSA’s (2014) model explains this value as a need to move past cultural stereotypes and biases, provide gender-affirming services, integrate traditional cultural wisdom, and provide care that is responsive to the racial, ethnic, and cultural needs of communities served. This value also includes an acknowledgment of the historical trauma faced by certain racial and ethnic groups (SAMHSA, 2014). A focus on cultural responsiveness, however, does not encompass the nuances we found in the data. SSW respondents called for increased focus on diversity, equity, and inclusion; racial and cultural sensitivity training; and measurable anti-racism action in response to the pandemic and 2020 racial reckoning. SSWs seemed to expand upon the ideal of cultural responsiveness to include action toward equity and anti-racist practice. Given that anti-racism is not specifically encompassed in the current conceptual framework for a trauma-informed approach, this may be an area for further development of the model. Prior research has shown that educators conceptualize cultural responsiveness and anti-racism differently and thus approach their practice differently, according to which goal they are attempting to achieve (Galloway et al., 2019). For practitioners, we recommend continued assessment of how best to support the needs of students and families with diverse backgrounds, including a concerted effort to involve school constituents in identifying culturally responsive and anti-racist approaches to trauma along with recruiting educators and mental health professionals with diverse backgrounds and perspectives. We also suggest research that explores outcomes associated with cultural responsiveness and anti-racism as part of trauma-informed approaches in schools.

#### Essential Facets of a Trauma-Informed Response Highlighted by School Social Workers

SSWs suggested a variety of school-based responses to the trauma students, families, and staff experienced during the early phase of the COVID-19 pandemic. These included universal trauma training for staff, increased organizational capacity and resources to address student and staff mental health, prioritizing social-emotional needs upon return to school, and the integration of SSW voices in schoolwide administrative responses. Some of these recommendations relate directly to existing conceptualizations for a trauma-informed school. For example, universal trauma training is typically recommended to meet the goal of increasing staff recognition of the impact of trauma and enabling them to recognize trauma reactions in themselves and others (Cole et al., 2013; MPS, 2020; NCTSN, 2017). SSWs also suggested a need for schoolwide responses and highlighted why SSWs should be involved in administrative decision making. Without SSWs’ socioecological and organizational focus, schools’ post-pandemic responses may be limited to classroom discussions about emotional regulation and social-emotional skills or individual counseling for students manifesting disruptive behaviors in class rather than a comprehensive, whole-school response to meeting the needs of staff, students, and families.

#### Challenges of Meeting Varied Definitions of Safety During the Pandemic

SSWs conceptualized school safety in a variety of ways, including physically, emotionally, and psychologically. As such, they had differing opinions on how best to respond to safety needs, whether to reopen schools quickly to promote social-emotional well-being of students or to keep schools closed with remote instruction to ensure the physical safety of children who could not be vaccinated or immunocompromised staff. Some understood the complexities and tradeoffs of different conceptualizations of safety. We believe there are two key takeaways from these findings. First, responses demonstrated that school safety must include emotional *and* physical well-being. Second, the pandemic elevated awareness of school safety to an organizational level. Traditionally, SSWs and other mental health professionals have viewed safety during trauma recovery through an interpersonal lens (e.g., Keeshin & Strawn, 2014). The global pandemic illustrated that interpersonal safety is insufficient to keep students, families, and staff safe; thus, safety in a post-pandemic world requires commitment by the whole school organization, further highlighting the necessity of involving mental health professionals in an integrated response.

#### Differing Views Regarding the Importance of Relationships, Depending on Constituent of Interest

SSWs emphasized trust in developing and sustaining positive relationships with students and families and the importance of working collaboratively among their school colleagues, but they did not mention trust as a contributing factor to collaboration with school colleagues. We see reflected in the qualitative responses that SSWs believed student and parent participation requires trust. We do not know whether SSWs also believe trust is essential for effective collaboration between colleagues; however, we know that SSWs valued their relationships with other staff, and that the literature suggests effective collaboration requires trust (SAMHSA, 2014). Based on the available literature (e.g., Cole et al., 2013; Harris & Fallot, 2001; SAMHSA, 2014), it is clear that trust both facilitates and is a positive side effect of healthy relationships in a trauma-informed approach. However, the lack of consistency in reporting the value of trust and collaboration across all constituents in our findings suggests that SSW practitioners should reconsider the way they are thinking about trust and collaboration across various school constituents.

A third characteristic of positive, school-based relationships highlighted in the trauma-informed organizational literature is empowerment or giving voice to constituents (e.g., Harris & Fallot, 2001; SAMHSA, 2014; Wolpow et al., 2009). In our findings, SSWs spoke of the importance of empowering students and families while also lamenting their own feelings of disempowerment or lack of a voice in organizational decision making during the COVID-19 pandemic. From an organizational perspective, this is a very challenging circumstance. It might be said that educators and other school staff cannot provide for students what they do not experience. We know from the literature that trust between teachers and school administrators has an effect on school climate and perceptions of principal leadership quality (Tschannen-Moran & Gareis, 2015). As a result, it appears that everyone—from school administrators to bus drivers—must be involved in the creation and sustainment of a trauma-informed environment and benefit from it (SAMHSA, 2014).

### Practice and Policy Implications

Findings have several practice and policy implications as schools reopen for in-person instruction and adapt to meet the heightened needs of students, staff, and families.

#### Increased Funding Translates to Increased Opportunities and Challenges

Early in the COVID-19 pandemic, non-academic school personnel, including social workers, reported layoffs and feared future cuts (Mahnken, 2020; Paltrow, 2020). Cuts to education were ultimately less severe than expected, however, due to the federal Coronavirus Aid, Relief, and Economic Security Act and reallocation of other funding (Griffith & Berry, 2020). In total, more than $200 billion have been committed to K-12 public education since the start of the pandemic (Griffith, 2021). The 2020 police murders of Breonna Taylor, George Floyd, and others sparked a national movement to defund police and reallocate funding toward the hiring of counselors, social workers, and other mental health professionals (Jones, 2020). These calls were generally coupled with a goal to support students’ mental health and address potentially traumatic experiences suffered during the pandemic (Jones, 2020, 2021). New funding was targeted toward low-income schools and communities; however, much of it was a one-time allocation (Fensterwald, 2021). These funding infusions and calls for change provide tremendous opportunity to increase equity in schools and support our most disadvantaged communities, but they also create uncertainty about how to use funds wisely to sustain long-term gains.

#### Staffing Shortages and the Need for a Stronger Pipeline of School Professionals

At the same time that schools are attempting to hire mental health professionals to respond to the heightened needs of children and families (AAP, 2021; Kelly et al., 2020a; de Miranda et al., 2020; Lee, 2020), many such positions are going unfilled (Chiriguayo, 2021). This staffing shortfall is part of a larger national trend of shortages for teachers, bus drivers, custodians, and other school staff (Associated Press, 2021; Heyward, 2021). Carver-Thomas et al. (2021) made several recommendations to strengthen the educator workforce: invest in pathways to develop and retain teachers, particularly those of color; increase financial support for teacher candidates; streamline licensing requirements; increase substitute teaching rates to attract qualified candidates; and invest in professional development. Many of these recommendations apply to the recruitment and retention of other skilled school staff, including social workers, counselors, and psychologists. They also relate to policy recommendations we made immediately following our national survey, which included a need for an expanded school mental health workforce, increased social-emotional supports for staff, modifications to licensing requirements, and increased professional development related to pandemic service delivery (Kelly et al., 2020b).

#### A Need for Greater Integration of Whole-School, Trauma-Informed Approaches

Despite legislative support for trauma-informed approaches in schools (see the Every Student Succeeds Act of 2015 and SUPPORT for Patients and Communities Act of 2018), such approaches have not yet become well-integrated into many schools’ missions or practices (Maynard et al., 2019; Thomas et al., 2019). As our findings show, whole-school, trauma-informed approaches require the support of a school’s administration and staff beyond its mental health professionals. Unfortunately, existing models for trauma-informed organizations do not make clear who should be responsible for leading trauma-informed organizational adaptations (e.g., Cole et al., 2013; SAMHSA, 2014). As shown by our findings, SSWs are well-suited to lead implementation of trauma-informed approaches in schools due to their socioecological lens.

## Strengths, Limitations, and Future Research

Our findings should be understood in relation to their strengths and limitations. This study benefited from the collection of 115 single-spaced pages of detailed written responses to open-ended survey questions from practicing SSWs in the early phase of COVID-19. The gender and racial/ethnic composition of study participants reflect prior workforce surveys (Kelly et al., 2010, 2015; Salsberg et al., 2017). Participants represented almost all of the states where SSWs work, but this was not intended to be a nationally representative sample and, thus, we believe there was self-selection among respondents. Responses to open-ended survey questions were limited to a small proportion of the quantitative study sample (approximately one-third) and the majority of respondents only answered two open-ended questions included in the original survey. Just 72 respondents answered follow-up questions sent in July 2020. Although we do not have demographic information for follow-up respondents, their personal and professional characteristics almost certainly influenced their decision to respond. Also, it is worth noting that this study was opportunistic in that it was formulated in response to thematic trends we saw during initial inductive coding of SSWs’ written responses to a COVID-related survey (see Capp et al., 2021). The research project was not designed to learn about trauma-informed approaches in schools; therefore, any relevant framework could have been applied to the data (e.g., positive school climate, SEL, etc.). Future research is thus needed to ascertain how SSWs perceive conceptual frameworks for a trauma-informed approach in relation to their role and how the process of developing trauma-informed approaches is unfolding in their schools.

As noted in the discussion, future research is also suggested to determine how SSWs view their role in relation to the avoidance of retraumatization (component seven of a trauma-informed approach) and the outcomes expected from cultural responsiveness and anti-racist practice as part of a trauma-informed school. Future research should include epidemiological approaches to ascertain what a nationally representative sample of SSWs experienced during the pandemic and look into patterns of regionality. Additional research is also needed to better understand how SSWs are responding to the potentially traumatizing experiences that staff and students faced during the pandemic after a full year of school disruptions, and to assess the process of reopening schools in ways that address trauma, equity, and safety for all constituents.

## Conclusion

The COVID-19 pandemic and resulting school disruptions provided a unique opportunity to understand how school social workers perceived the mental health and trauma-related needs of school constituents, and how those views related to existing models for trauma-informed approaches in schools. SSWs viewed summer 2020 as a potentially traumatic period that required a trauma-informed response by our nation’s schools. SSWs made several recommendations related to implementing a trauma-informed response, including ensuring the psychological, emotional, and physical safety of school constituents; promoting and sustaining strong school-based relationships; and prioritizing mental health supports for students and staff. Suggestions reflected a whole-school approach to trauma, which requires the involvement of all school staff. Concurrently, suggestions shed light on aspects of a whole-school, trauma-informed response that may be missing from existing conceptual frameworks, for example, a measurable anti-racist agenda in keeping with SSWs’ commitment to social justice (NASW, 2021). SSWs’ views are particularly salient given the new resources available for schools due to the CARES Act, American Rescue Plan of 2021, and calls to defund school police in favor of hiring more social workers and implementing trauma-informed approaches (Griffith, 2021; Griffith & Berry, 2020; Jones, 2020, 2021).

## Data Availability

Not applicable
